# Role of topical application of gentamicin containing collagen implants in cardiac surgery

**DOI:** 10.1186/1749-8090-9-122

**Published:** 2014-07-08

**Authors:** Pankaj Kumar Mishra, Ahmed Ashoub, Kareem Salhiyyah, Dincer Aktuerk, Sunil Ohri, Shahzad G Raja, Heyman Luckraz

**Affiliations:** 1 Cardiothoracic Unit, Heart and Lung Centre, Wednesfield Road, Wolverhampton WV10 0QP, UK; 2 Wessex Cardiothoracic Centre, Tremona Road, Southampton SO16 6Y, UK; 3 Harefield Hospital, Hill End Road, London UB9 6JH, UK

**Keywords:** Surgical site infection, Supercial sternal wound infection, Deep sternal wound infection, Mediastinitis, Topical gentamicin, Gentamycin containing collagen implants, Collatamp

## Abstract

Sternal wound infections (SWI) continue to be a major cause of concern after cardiac surgery. It leads to prolonged hospital stay and increased morbidity, mortality and increased hospital costs. Prophylactic systemic antibiotics have been used to prevent surgical site infection (SSI). However, prolonged postoperative use of systemic antibiotics can lead to emergence of resistant organisms. Gentamycin Containing Collagen Implants (GCCI) when used during sternotomy closure produces high local antibiotic concentrations in the wound with a low serum concentration. There is evidence that the concentration of gentamicin in the mediastinal fluid reaches levels high enough to be effective against bacteria that are considered resistant to gentamycin and other antibiotics.

However, questions have been raised about the safety and efficacy of GCCI. There were concerns whether GCCI can lead to systemic absorption with renal impairment and whether use of topical antibiotics can lead to emergence of antimicrobial resistance.

We, hereby, review the literature on GCCI (Collatamp) and take the opportunity to appraise the scientific community about their role in cardiac surgery. Several recent studies have supported their clinical effectiveness. They should be used in dry condition and should not be soaked in saline even for a short period prior to use. However, for GCCI to become part of routine practice in cardiac surgery further large randomised studies are required. As the incidence of sternal wound infection is low in the specialty of cardiac surgery, for any study to be sufficiently powered to address this issue, multicenter studies might be the way forward.

Based on the evidence presented in this manuscript it is recommended GCCI (Collatamp) can be a cost effective adjunct for prevention of sternal wound infection. They can also be used for treatment of Deep Sternal Wound Infection.

## Review

Surgical Site Infections (SSI) is associated with increased length of hospital stay and cost of care [1]. Sternal wound infection (SWI) after cardiac surgery continues to be one of the most serious postoperative complications [2-6]. Superficial Sternal Wound infection (SSWI) leads to increased morbidity while Deep Sternal Wound Infection (DSWI) after cardiac surgery is a serious complication causing substantial increase in both morbidity and mortality. Efforts have been made to decrease the incidence to SWI. However, despite all efforts, DSWI is an everyday challenge in the life of a cardiothoracic surgeon.

In the last decade, new ways have been investigated to reduce the incidence of SSWI and DSWI. After positive experience in other specialties (e.g. colorectal surgery, orthopaedics) with topical antibiotic preparations, resorbable gentamicin-containing collagen implant (GCCI) has been introduced in cardiac surgery in an effort to reduce the incidence of sternal wound complications [7-9].

Gentamicin Containing Collagen Implant (GCCI) delivers high local concentrations of gentamicin with low serum levels [2-4]. The high local antibiotic concentrations may have an effect on bacteria that are normally considered to be resistant [2,10]. Although gentamicin is generally used for gram-negative infections, it does have a spectrum of bactericidal activity for many gram-positive organisms, including staphylococci [11].

Gentamicin Containing Collagen Implant (GCCI) are licensed for use in cardiac surgery in over 50 countries but their effectiveness at preventing sternal wound infections (SWIs) continues to be debated [1,2,12]. We hereby, review the published clinical data for prophylactic application of resorbable GCCI following cardiac surgery. We take the opportunity to appraise the scientific community about the safety and efficacy of GCCI in prevention of postoperative sternal wound complication.

### Definition and classification of SSI

The criteria for definition and classification of Surgical Site Infections (SSI) in the literature vary. A majority of studies have used criteria laid down by Centers of Disease Control and prevention for surgical site infection [2,13,14]. It is focused on the depth, but not on the clinical severity, of the infection [13]. In UK NICE (National Institute of Clinical Excellence) has issued guidelines for recognition and management of SSI [15]. Deep SWI includes all SWI with sternal dehiscence or infections down to the sternum, even when the sternum remained stable [2,13]. Infections are usually classified as definite if both clinical signs of infection and prespecified bacterial cultures were positive [1,2,13]. A more comprehensive clinical wound scoring system, such as the ASEPSIS score will be more informative but more laborious to use [16,17].

### Pathophysiology of Sternal Wound Infection (SWI)

#### Incidence and Risk factors

The reported incidence of postoperative SWI varies considerably; because of differences in definitions and classification of infections and variations in follow-up [2,18-27]. Sternal wound infection (SWI) has a reported overall incidence between 0.5% and 6% [1]. However, in high-risk patients the incidence has been estimated at between 12 and 20% with an associated mortality rate between 14% and 47% [7,18-22]. Schersten et al. suggest that the incidence of SSI goes higher in studies reporting surgery on high-risk patients and also in patients undergoing emergency operations [28].

Sternal wound infections can be divided into superficial and deep infections (DSWI) [23]. Sternal infections can also be classified as early and late [23,26]. Late infections include osteomyelitis, subcutaneous abscess, sterno-cutaneous fistulas and mediastinitis [23]. In cardiac surgery, numerous risk factors for SSI exist, such as obesity, diabetes, COPD, re-operation, use of two internal mammary arteries, and duration of surgery [7,25,29-32]. In the presence of bacterial contamination a slight instability in an osteal fixation promotes the development of clinical infection [33,34].

#### Bacteriology

The most common bacteria involved in postoperative wound infections are Gram-positive cocci, mainly staphylococci— either coagulase-negative staphylococci (CoNS) or Staphylococcus aureus [35-37].

Coagulase-negative staphylococci (CoNS) (usually S. epidermidis) have become the most common cause of SWI in many of the reported series [33]. CoNS were also the most commonly present agent in cases with multiple bacterial agents [33-35]. 80% were resistant to aminoglycosides and 81% were resistant to methicillin [33]. CoNS often cause chronic infections by forming a biofilm through attachment to an implanted device such as steel wires following median sternotomy [38]. Other organisms e.g. Streptococci, Gram-negative bacteria and fungi, especially Candida albicans, can also cause SWI [39,40].

### Prevention of DSWI

Techniques to decrease SSI include preoperative skin care, aseptic surgical technique, gentle tissue handling and perioperative antibiotic cover [41-43]. Intravenous (IV) antibiotic prophylaxis for median sternotomy has been clearly shown to reduce the wound infection rate in several studies and is a routine practice in most cardiothoracic units [27]. There is some evidence that rigid sternal fixation with, usage of seven or more single sternal fixation wires leads to a lower rate of SWI, compared with only six single wires [33,44].

#### Topical antibiotics and GCCI

Gentamicin is predominantly used for gram-negative infections. However, it does have a spectrum of bactericidal activity for many gram-positive organisms, including staphylococci [2,45-48]. Administration of systemic antibiotics may lead to a greater risk of antibiotic resistance [46-48]. The emergence of local antibiotic-eluting products such as resorbable gentamicin-containing collagen implant (GCCI) enables delivery of high local concentrations of gentamicin with corresponding low serum levels. The use of collagen as a carrier also has a positive effect on wound healing [33].

GCCI sponges were introduced in 1985 for the prevention of surgical site infection and they were mainly used after laparotomy [49]. GCCI can be an effective adjunct in reducing the rate of SSI following cardiac surgery particularly in high risk patients [50]. GCCI may also have a role to play in the treatment of established DSWI [51]. However, currently there is no consensus about their use in patients undergoing cardiac surgery [49].

#### Collatamp

Collatamp® (Collatamp® G EUSA Pharma Europe) is a lyophilised collagen delivery system for gentamicin, used in the prevention and treatment of SSI [52]. Bovine collagen is rapidly absorbed and is an efficient vehicle for local antibiotics delivery. The implants are degraded by collagenases, and do not need to be surgically removed [53]. Collatamp® delivers a high dose of gentamicin, the level and speed of which is dependent on the local blood flow at the site of the wound [54]. The antibiotic is concentrated locally in the tissue and does not reach significant serum levels, which reduces the risk of systemic side effects (ototoxicity/nephrotoxicity) [54]. Collatamp® also accelerates haemostasis and positively influences wound healing [53,54]. There is evidence that the concentration of gentamicin in the mediastinal fluid reaches levels high enough to be effective against bacteria that are normally considered resistant (including most CoNS) [2,10,51].

### Mechanism of action and pharmacokinetics

Synthetic collagen-based products have been used for their angiogenic properties and their role as a matrix or “scaffold” to promote fibroblast migration and granulation tissue formation [55-59]. Collatamp helps local haemostasis and has been advocated for use in areas with seeping haemorrhage with a high risk of infection. Haemostasis is triggered when blood comes into contact with exposed endogenous collagen fibrils or renatured collagen fibrils like those in Collatamp. The adhesion and aggregation of platelets is induced on the collagen fibrils of Collatamp and the coagulation process is accelerated. The sponge-like structure of Collatamp stabilises the wound clot and also promotes granulation and epithelialisation. Overall effect is accelerated granulation tissue formation and enhanced healing process [51,60,61].

Systemically administered, gentamicin may be toxic [7,62]. However, locally administered, the serum concentrations remain well below toxic levels [7,53,62]. The pathogens involved in SSIs after cardiac surgery is mainly coagulase-negative staphylococci (Staph Epidermidis) and Staph aureus, especially in deep infections [4,7,29]. Gentamicin has good activity on these organisms [7,53].

The bactericidal effect of aminoglycosides is dependent on the peak level, and a high peak level is associated with a high bactericidal effect [51,63,64]. The minimum inhibitory concentration (MIC) of gentamicin for systemic antibiotic therapy is 4 mg/L. Leyh et al. showed that with GCCI high local gentamicin levels (>300 mg/L) for 36 h are detected in mediastinal effusions [50]. Serum concentrations remain low (1–4 mg/L 1 h postoperatively and ≥1.5 mg/L after 24 h). These values are well below the toxic threshold (10 mg/L) [51,53].

Therefore, a high bactericidal concentration of gentamicin in the anterior mediastinum and sternal bone can be anticipated. Besides, resistance is not an absolute feature of bacteria, but it results from the relation of growth inhibitory concentration of the bacteria to inhibitory concentration in the tissue. Grimm et al. demonstrated that bacteria which are resistant at the minimal inhibitory concentration (MIC) level are sensitive to higher gentamicin levels [65]. The peak local levels of gentamicin with GCCI are 75–200 times higher than the MIC making it highly effective against even resistant isolates [51]. In the study of Leyh et al., high bactericidal gentamicin levels were detected for 36 h after surgery [51]. It is uncertain how long gentamicin remains in mediastinal tissues, but release is dependent on the local blood flow [51].

Gentamicin kills bacteria by inhibiting protein synthesis and destabilizes bilayered membranes of bacteria [7]. Thus, each component of collagenous gentamycin may be effective, whereby gentamicin acts as a bactericidal agent and the carrier substance collagen possibly supports the wound healing process and bone regeneration [7,51].

Initially concerns were raised that introduction of a topical antibiotic prophylaxis could induce antibiotic resistance with subsequent reduced effect of the prophylaxis [33]. However, pharmacokinetics of topical use of Collatamp shows an early high peak in the local gentamicin concentration, low serum concentrations and then rapid disappearance of the drug which reduces the selection of resistant bacteria. This pharmacokinetic profile is even favourable compared to normal IV use [2,10].

### Use of collatamp in cardiac surgery

Collatamp is available in 3 different presentations: 5 cm × 5 cm; 10 cm × 10 cm and 5 cm × 20 cm sizes [66]. Collatamp® implants are wrapped around the sternal edges prior to wound closure to ensure maximal concentration of gentamicin on all sides of the wound and inside the bone marrow. Sternal wires are used along the length of the sternum to firmly close the wound. Though collagen is rapidly resorbed, earlier it was argued that placing the collagen sponges between the sternal halves, rather than behind the sternum, might affect the sternal healing. Friberg et al. did not identify any such adverse effect [2,10,33,44]. However, the authors have emphasised the importance of a rigid fixation (defined as at least seven single sternal fixation wires), with complete compression of the sponges], to achieve a maximal reduction in deep infections by the collagen-gentamicin implant [44]. Too thick layer of Collatamp should be avoided as it can promote sternal instability [28].

Collatamp strips can also be used in vein harvesting site closure. Collatamp can be cut to size to fit the area to be treated. For SVG (Saphenous Vein Graft) harvesting site, once the vein has been removed, one 5 × 20 cm Collatamp® implant can be folded and packed into the open wound. The wound is then closed with sutures.

However, the patient’s body weight and the total amount of gentamicin should be taken into account. When using for both sternotomy and vein harvest site closure the number and size of the implants should be selected so that a total dose of 9 mg gentamicin sulphate per kg body weight is not exceeded [66]. Collatamp® should be stored between 4°C and 25°C [66].

### Cautions and contraindications

Collatamp® is approved for commercial use within the European Union. No evidence of allergic reactions or adverse local effects on sternal healing has been reported [2]. In one study a higher incidence of early reoperation for bleeding in the Collatamp group was reported [2]. No side effects have been reported to date [2,43,50,51]. If the recommended maximum dose is exceeded, gentamicin-specific side effects cannot be ruled out completely, especially in the case of renal failure [66].

Systemically effective therapeutic blood or plasma levels are not generally achieved with the use of Collatamp®. However, interactions related to gentamicin should be considered. Besides, in patients with impaired renal function, the benefits of Collatamp® must be carefully considered [2,10,12,66].

Collatamp should not be used if a protein allergy is known or intolerability towards gentamicin has been observed. No experience has been gained in use during pregnancy and breast-feeding [66]. For this reason, the indication should be strictly established during pregnancy and breast-feeding [66].

No interactions have been reported to date. If adjuvant systemic treatment with gentamicin, other aminoglycoside antibiotics or other ototoxic or nephrotoxic drugs is necessary, the cumulative effects should be taken into account. In general, the number and size of the sponges should be selected so that a total dose of 9 mg gentamicin sulphate per kg body weight is not exceeded [66].

### Cost analysis

DSWI are the most common cause of prolonged hospital stay and increased hospital costs [7,19-22,50,51]. For GCCI to become part of routine practice clinical effectiveness has to be matched with cost effectiveness [12]. The use of Collatamp as an adjunct to IV antibiotics was found to be cost saving step [2,44]. This was due to fewer wound infections and lower costs involved in subsequent treatment despite the cost of the implants [2,44]. This was particularly relevant for high-risk patients e.g. those with diabetes or BMI > 25 kg/m2 [44]. CoNS infections have an insidious presentation. They are difficult to treat, requiring prolonged courses of antibiotics, often requiring extensive surgical debridement with the use of muscle flaps [2,67]. It results in consumption of substantial healthcare resources [2,67].

Eklund et al. suggests that it would be economical to use gentamicin-collagen implants in every CABG patient, since the treatment of mediastinitis is extremely expensive [7,50,51]. Friberg et al. concluded that despite the high cost of the gentamicin impregnated sponges, the use of two sponges, in addition to intravenous antibiotic prophylaxis, was cost effective, resulting in both lower costs and fewer infections for all patients as well as for high risk patients [2,44].

### Clinical effectiveness: literature review

A wealth of literature is now available on GCCI and Collatamp®. Friberg et al. (Table 1) published a large double blind RCT (LOGIP Trial) on the role of GCCI in cardiac surgery [2]. Sternal closure was performed by the senior members of the team i.e. the operating surgeon. This study demonstrated an overall relative risk reduction in SSI in the GCCI-treated patients compared to the control group. The GCCI group demonstrated reduced need for surgical revision (2.3% vs. 4.0%) and postoperative IV antibiotic usage (11.6% vs. 18.0%) [2]. Use of GCCI was shown to be even more beneficial in preventing both SSWI and DSWI in high-risk patients. In obese patients the difference in DSWI did not attend statistical significance. No significant difference in postoperative renal function or 60 day mortality was found. There was also no indication of any increase in the occurrence of gentamicin-resistant isolates. An unexplained finding was the significantly higher rebleeding rate in the GCCI arm [2]. The authors suggested that the incidence of reoperation for bleeding in the GCCI group was similar to that seen in routine practice, and that the re-operation rate in the control group was lower than expected [2].

**Table 1:** A summary of important studies on the use of GCCI (Gentamycin Containing Collagen Implants) in cardiac surgery

**Authors**	**Study design, n = Number of Subjects**	**Treatment groups**	**Results**
Friberg O et al. [2], 2005 LOGIP Trial	Double blind, Randomized, controlled, two-centre study. Patients undergoing cardiac surgery through median sternotomy - including operations in the ascending aorta.	n = 1950 total patients	Wound infection (<2 months post-operatively):
		Treatment Gr (Gr I): Collatamp between the Sternal edges (n = 983)	Group I vs Group II 4.3% vs 9.0% (RR 0.47; p < 0.001)
		Control Gr (Gr II): Standard closure (n = 967)	Early reoperation for bleeding was more common in the treatment group (4.0% vs 2.3%, p = 0.03).
	N = 1950	Standard antibiotic prophylaxis given to both groups	
			Need for postoperative antibiotic treatment: Group I: 11.6% vs Group II: 18.0% (RR 0.64: p < 0.001)
	Evaluation: Double blind, ITT		
Friberg O et al. [33], 2009	Prospective study,	n = 2326 total patients	Wound infection (<60 days postoperative):
	Two centre study		
	(Control Gr from LOGIP Trial)	Treatment Gr (Gr I): n = 1359	Group I vs Gr II 3.7% vs 9.0% p < 0.001
		Control Gr (Gr II): n = 967	Surgical revision: Group I vs Group II 1.8% vs 3.9% (p < 0.001)
		Standard antibiotic prophylaxis given to both groups	
Eklund et al. [7], 2005	Randomised, Controlled Trial, Single-centre study.	n = 542 total patients	Wound infection (<3 months post-operatively):
		Treatment Gr (Gr I): n = 272	
			Group I vs Group II 4.0% vs. 5.9% (p = n.s.)
		Control Gr (Gr II):	
	Evaluation: Partially Blinded	n = 270	Incidence of mediastinitis: 1.1% vs 1.9% (P value = NS)
		Standard antibiotic prophylaxis given to both groups. Any patient staying in hospital for >72 hrs received IV Vancomycin in addition to routine IV Cefuroxime.	
Schersten et al. [28], 2007	Prospective Study with historical controls	n = 2026 total patients	Wound infection (mediastinitis): Group I vs Group II
		Treatment Gr (Gr I): n = 1091	
		Control Gr (Gr II):	0.75% vs 1.9%
		n = 935	(p < 0.05)
		Standard antibiotic prophylaxis given to both groups	
Leyh et al. [51], 1999	Observational Study	N = 42 Patients of DSWI after cardiac surgery were treated with Collatamp with or without other surgical interventions	No definite conclusion regarding direct benefit of Collatamp use can be drawn from this study.
	(No Control Group) Impact of GCCI on treatment of DSWI, to assess side effects of Gentamycin topical use, and Gentamycin		
			High (bactericidal) local levels of Gentamycin noted in mediastinal fluid
	level in mediastinal fluid		
Bennett-Guerrero et al. [67], 2010	Randomised, Controlled Trial, Single blind,	n = 1502 total patients	Wound infection (<90 days post-operatively):
		Patients with diabetes n = 1006 [67%]	Incidence of all types of wound infection Group I Vs Gr II 8.4% vs 8.7% (p value = n.s.)
	Multicentre study	Patients with BMI > 30, n = 1137 [76%]	
	Patients undergoing cardiac surgery and at high-risk for sternal wound infection (diabetes, BMI > 30 or both)		
			Incidence of DSWI Group I: 1.9% vs. 2.5% (p value = n.s.)
		Treatment Gr (Gr I): n = 753	
		Control Gr (Gr II):	
		n = 749	
		Standard antibiotic prophylaxis given to both groups	
			Incidence of SSWI Group I: 6.5% vs. 6.1% (p value = n.s.)
			Re-hospitalisation for sternal wound infection (<90 days post-operatively): Group I: 3.1% vs 3.2% (p value = n.s.)
Birgand et al. [68], 2013	Quasi-experimental prospective study (single-centre)	n = 552 total patients Intervention period	DSWI incidence rate: Intervention period
		patients managed with GCCI n = 175	patients managed with GCCI: 12.6%
	Diabetic and/or overweight patients undergoing CABG with bilateral internal mammary artery grafts.		
		Intervention period patients managed without GCCI n = 88	Intervention period patients managed without GCCI: 6.8%
		Retrospective Control Gr, preintervention era group n = 289	Retrospective Control Gr, preintervention era group: 13.8%
			No statistically significant differences between three groups.
			The group managed with the sponge had a higher proportion of gentamicin-resistant micro-organisms.
		The end-point was the rate of reoperation for deep sternal wound infection.	
Cohen et al. [73], 2010	Retrospective Case series	n = 216 total patients	Wound infection: Group I vs Gr II: 0.0% vs 9.0% (p value = 0.0220).
		Treatment Gr (Gr I): n = 108	
		Control Gr (Gr II):	
		n = 108	
Raja et al. [50], 2011	Patient case series	n = 194 total patients	Wound infection: Incidence of SSWI Group I vs Gr II: 2.1% vs 6.2% (p value = 0.01). Incidence of DSWI Group I vs Gr II: 2.1% vs 3.1% (p value = n.s)
	Patients undergoing cardiac surgery via sternotomy	Treatment Gr (Gr I): n = 97	
		Control Gr (Gr II):	
		n = 97	
		Standard antibiotic prophylaxis given to both groups	
Schimmer et al. [74], 2012	Randomised, Controlled Trial, Double blind	n = 723 total patients	Wound infection (<30 days):
		Treatment Gr (GrI) GCCI Gr, Collagen Implant with Gentamycin: n = 354	
			Incidence of SSWI/DSWI
	Single-centre study		
			(Group I vs Gr II)
			0.56%/1.9% vs
			3.52%/2.9%
			(p = 0.013)
	Comparison of a GCCI versus a simple Collagen sponge	Control Gr (Gr II) Simple collagen implant without Gentamycin:	
		n = 369	
		Standard antibiotic prophylaxis given to both groups	
Creanor et al. [12], 2012	Meta-analysis of randomised controlled trials	Three randomised controlled trials (published between 2005 and 2010) involving 3,994 patients	There is insufficient evidence of the effectiveness (or otherwise) of GCCI in preventing SWIs following cardiac surgery. However, some evidence does exist that such sponges can reduce the incidence of deep infections in high risk patients
Chang et al. [75], 2013	Systematic Review and Meta-analysis of Randomized Trials	Fifteen randomised controlled trials involving 6979 patients	Use of GCICI was associated with a significant decrease in SSI with an NNT of 21 p = 0.001;
			Post hoc analysis showed that GCCI implants are effective in reducing SSI in sternotomy wounds. (OR = 0.59; 95% CI: 0.37–0.96;) (P = 0.03; NNT = 32)
Mavros et al. [76], 2012	Systematic Review and Meta-analysis of Randomized Trials	Four RCTS involving 4672 patients	GCCI reduced the risk of DSWI and need for surgical revision. No impact on SSWI or all cause mortality. Most commonly isolated pathogens were CoNS.

Treatment Group = GCCI Group.

RCT = Randomised Controlled Trial.

NNT = Numbers Needed to Treat.

SSWI = Superficial Sternal Wound Infection.

DSWI = Deep Sternal Wound Infection.

SSI = Surgical Site Infection.

SWI = Sternal Wound Infection.

CoNS = Coagulase Negative Staphylococcus Aureus.

CABG = Coronary Artery Bypass Graft.

BMI = Body Mass Index.

RR = Relative Risk.

OR = Odds Ratio.

CI = Confidence Interval.

NICE guidelines 2008 acknowledged the role of GCCI in ‘Prevention and treatment of surgical site infection’ based on the LOGIP trial [15]. However, it emphasised the need for evaluation of the long-term effects on microbial resistance [15].

For the two centres enrolling patients in LOGIP Trial use of GCCI became a routine practice. As suggested by NICE guidelines, the authors re-evaluated their technique with a prospective non randomised study designed for comparison with the previous control group from the LOGIP trial [33] (Table 1). The highly significant risk reduction found in the previous trial at the same institute raised ethical concerns in randomizing patients again to control/placebo [33]. They also changed the surgical technique and use of at least seven single wires for sternal fixation was emphasized in the protocol of this second study (compared to 6–8 wires used at Surgeon’s discretion in the LOGIP Trial) [2,33]. The incidences of both superficial and deep SWI were less than half of that in the control group. The incidence of SWI was slightly lower than that in the treatment group of the primary LOGIP trial (4.3% SWI with 2.3% deep SWI) probably due to improvement in surgical technique. However, it is not possible to differentiate the relative effects of each of the variables (improvement in surgical technique and impact of GCCI) in improvement of sternal wound complications [33].

No trend towards less effect over time could be detected after 7 years of daily use [2,33]. A microbiological analysis between the two studies after a gap of several years showed that there had been no change in types of causative bacteria and no absolute increase in SSIs caused by aminoglycoside resistant microbes over time [2,33]. The organisms isolated were predominantly CoNS. They were resistant to both aminoglycosides and methicillin in approximately 80% of cases. These ‘resistant’ strains may be susceptible to the extremely high local concentrations achieved with GCCI [33].

Eklund et al. performed a RCT which did not show any evidence of beneficial effect of GCCI in prevention of sternal wound infection [7] (Table 1). However, the authors emphasised that with the infection rates in their study (4% study group; 5.9% control group), the sample size necessary to detect a significant (*P* < 0.05) RR with a statistical power of 0.8 would be 1275 patients in each group. The authors acknowledged that the study was too small to draw any firm conclusions (total 542 patients with 272 and 270 patients in each group).

Schersten et al. reported that after adding GCCI to their standard treatment protocol in a consecutive and unselected series of 1091 patients, they noticed a significant drop in the rate of mediastinitis [28] (Table 1). However, in this series of patients they made a significant change in their sternal closure technique (emphasis on more than 7 sternal wires for all patients) and it was difficult to discern the impact of GCCI alone in bringing down the rate of mediastinitis [28].

Leyh et al. treated mediastinitis with sternum refixation and gentamicin-collagen sponge successfully in 42 patients [51] (Table 1). The study was designed for effectiveness of measures to treat DSWI and not for prevention. Use of Collatamp resulted in high mediastinal fluid level of Gentamicin which will be bactericidal for even Gentamicin resistant strains (15% organisms were Gentamicin resistant in this study) [51]. However, as all patients were treated with multiple interventions, no definite conclusion can be drawn from this study regarding direct beneficial effect of GCCI use in treatment of DSWI [51].

Bennett-Guerrero conducted a multicenter RCT of 1502 patients in US for patients undergoing cardiac surgery and at high-risk for sternal wound infection (diabetes, BMI > 30 or both) [67] (Table 1). No significant difference was noted in the rates of SSWI or DSWI or rehospitalisation rate for wound infection up to 90 days after surgery [67]. Similarly, Birgand et al. conducted a quasi-experimental single-centre prospective study in diabetic and/or overweight patients undergoing coronary-artery bypass surgery with bilateral internal mammary artery grafts [68] (Table 1). They found no evidence of efficacy of GCCI [68]. Interestingly in both these studies GCCI were soaked in saline prior to use [67,68].

Corn T et al., Raja et al. and Friberg O et al. challenged the findings of these studies which were in contrast to the findings of several other contemporary studies [68-70]. The study protocol and the training video of the study by Bennett-Guerrero et al. describes dipping GCCI for 1–2 sec in saline prior to use [69-71]. The GCCI used in this trial is marketed by EUSA Pharma (Europe) which in their product information clearly states that the product should be used dry [66,69]. Gentamicin sulfate is highly soluble in aqueous solutions and dipping it in saline prior to use alters the release characteristics of Gentamicin decreasing its efficacy [66,69].

Lovering et al. conducted a study evaluating the impact of soaking gentamicin- containing collagen implants on potential antimicrobial efficacy of GCCI [72]. The study showed that even a short period of dipping of gentamicin-collagen sponge, before insertion into the patient, results in a significant loss of gentamicin [72]. The mean loss of Gentamicin was 6.7% at 2 s, increasing to 40.5% at 1 min and essentially total loss by 6 h of immersion [72]. This study provides clinching evidence that even a short period of dipping of Collatamp implants, before insertion into the patient results in a significant loss of gentamicin which may be of clinical significance [72].

In a retrospective review Cohen and colleagues compared a group of 108 patients who had received GCCI to a group of matched contemporary controls on a 1:1 basis [73] (Table 1). There was a significant difference in the incidence of post-operative SSI between the two groups (0% GCCI vs. 9% standard treatment; p value 0.022). Raja and colleagues conducted a propensity score analysis and compared the adjunctive use of GCCI in patients deemed at high risk of developing SSI to a group of matched controls [50] (Table 1). This study demonstrated a significant benefit of GCCI, which reduced the rate of superficial wound infection by 66% compared to standard treatment alone (2.1% GCCI vs. 6.2% standard treatment; p value 0.01) [50]. Although the patients in the GCCI group also had a lower rate of deep wound infection compared to the control group, this difference did not reach statistical significance [50].

Most of these studies compared GCCI versus routine antibiotic prophylaxis and suffered from a limitation as there were no control arms for Collagen sponge without Gentamycin. Schimmer et al. addressed this issue with a double blind RCT Comparison of a GCCI versus a simple Collagen sponge [74] (Table 1). The two types of sponges were implanted retrosternally in dry condition. This study has shown a significant reduction in DSWI and SSWI in patients undergoing median sternotomy for cardiac surgery with routinely application of a GCCI. Numbers needed to treat (NNT) for all sternal wound infections and deep sternal wound infections were 26 and 33, respectively [74].

Multiple randomized controlled trials (RCTs) produced conflicting results [74]. Creanor et al. performed a meta analysis of three randomised controlled trials (published between 2005 and 2010) involving 3,994 participants [12] (Table 1). Using random effects models, odds ratios (OR) and corresponding 95% confidence intervals (CI) were calculated for all SWIs and deep SWIs. There was insufficient evidence of the effectiveness (or otherwise) of gentamicin impregnated sponges in preventing SWIs following cardiac surgery. However, some evidence does exist that such sponges can reduce the incidence of deep infections in high risk patients. Chang et al. therefore conducted a systematic review and meta-analysis of all relevant RCTs [75] (Table 1). Fifteen RCTs encompassing a total of 6979 patients were included. The included studies were of moderate to high quality. Gentamicin-collagen implants significantly reduced SSI [OR = 0.51; 95% CI: 0.33–0.77; *P* = 0.001 [75].

Mavros et al. performed a meta-analysis of randomized controlled trials [76] (Table 1). Four RCTs were considered of high quality (score of 3 or more according to modified Jadad criteria) and were included in the study (4672 patients). GCCI reduced risk of DSWI (risk ratio, 0.62; 95% confidence interval, 0.39-0.97). However, no benefit was demonstrated regarding superficial sternal wound infections and all-cause mortality [76]. Pooled data from 2 randomized controlled trials (3410 patients), showed that use of GCCI also reduced the need for surgical revision of sternal wounds (risk ratio, 0.59; 95% confidence interval, 0.41-0.86). The most commonly isolated pathogens were coagulase-negative Staphylococcus spp (43%) and Staphylococcus aureus (28%) [76]. The authors concluded that there is large statistical heterogeneity among the existing trials and emphasised the need for additional large, high-quality randomized controlled trials [76].

Popescu et al. did a retrospective audit of 2238 patients where Collatamp was used in only 122 patients (5%) [77]. The rate of sternal wound infection in non-Collatamp group was 3% while it was 5% in Collatamp group [77]. The length of stay in hospital was shorter in Collatamp patients. No gentamicin sensitivity was recorded within the Collatamp group. Because of the small numbers and retrospective nature of the study no definite conclusion could be drawn [77].

It should be noted that in cardiothoracic surgery, where the infection rate is low, it is difficult to perform studies with appropriate statistical power. Eklund et al. highlighted that with the infection rates in their study (4% study group; 5.9% control group), the sample size necessary to detect a significant (*P* < 0.05) RR with a statistical power of 0.8 would be 1275 patients in each group [7]. As a result several studies are small, inadequately powered and it’s difficult to draw firm conclusions from such studies.

## Conclusion

Safety and efficacy of GCCI (Collatamp) has been debated and tested in several recent studies with conflicting results. Several recent studies have supported their clinical effectiveness. They should be not be soaked in saline even for a short period prior to use.

Based on the evidence presented in this manuscript, it is recommended GCCI (Collatamp) can be a cost effective adjunct for prevention of sternal wound infection. They can also be used for treatment of DSWI.

## Abbreviations

SWI: Sternal wound infections; SSI: Surgical site infection; GCCI: Gentamicin containing collagen implants; SSWI: Superficial sternal wound infection; DSWI: Deep Sternal wound infection; NICE: National institute of clinical excellence; CoNS: Coagulase-negative staphylococci; MIC: Minimum inhibitory concentration; RCT: Randomized controlled trials.

## Competing interests

The authors declare that they have no competing interests.

## Authors’ contributions

PKM: Literature search, preparation of the manuscript, revising the manuscript, submission, corresponding author. AA: Literature search, preparing the manuscript. KS: Literature search, preparation of the manuscript, DA: Literature search, preparation of the manuscript; SO: Literature search, preparation of the manuscript, Revising the manuscript; SGR: Literature search, preparation of the manuscript. HL: Literature search, preparation of the manuscript, revising the manuscript. All authors read and approved the final manuscript.
